# Enabling brain-wide mapping of directed functional connectivity at 3T via layer-dependent fMRI with draining-vein suppression

**DOI:** 10.1101/2023.10.24.563835

**Published:** 2023-10-29

**Authors:** Wei-Tang Chang, Weili Lin, Kelly S. Giovanello

**Affiliations:** 1 Biomedical Research Imaging Center, University of North Carolina at Chapel Hill, NC, USA; 2 Department of Radiology, University of North Carolina at Chapel Hill, NC, USA; 3 Department of Biomedical Engineering, University of North Carolina at Chapel Hill, NC, USA; 4 Department of Psychology & Neuroscience, University of North Carolina at Chapel Hill, NC, USA

## Abstract

Layer-dependent functional magnetic resonance imaging (fMRI) offers a compelling avenue for investigating directed functional connectivity (FC). To construct a comprehensive map of brain-wide directed FC, several technical criteria must be met, including sub-mm spatial resolution, adequate temporal resolution, functional sensitivity, global brain coverage, and high spatial specificity. Although gradient echo (GE)–based echo planar imaging (EPI) is commonly used for rapid fMRI acquisition, it faces significant challenges due to the draining-vein effect, particularly when utilizing blood oxygen level-dependent (BOLD) contrast. In this study, we mitigated this effect by incorporating velocity-nulling (VN) gradients into a GE-BOLD fMRI sequence, opting for a 3T magnetic field strength over 7T. We also integrated several advanced techniques, such as simultaneous multi-slice (SMS) acceleration and NORDIC denoising, to enhance temporal resolution, spatial coverage, and signal sensitivity. Collectively, the VN fMRI method exhibited notable spatial specificity, as evidenced by the identification of double-peak activation patterns within the primary motor cortex (M1) during a finger-tapping task. Additionally, the technique demonstrated BOLD sensitivity in the lateral geniculate nucleus (LGN). Furthermore, our VN fMRI technique displayed superior robustness when compared to conventional fMRI approaches across participants. Our findings of directed FC elucidate several layer-specific functional relationships between different brain regions and align closely with existing literature. Given the widespread availability of 3T scanners, this technical advancement has the potential for significant impact across multiple domains of neuroscience research.

## Introduction

Layer-dependent functional magnetic resonance imaging (layer-dependent fMRI) is an emerging field which measures the layer-specific activity noninvasively in humans. The ability to extract layer-specific signal provides an exciting opportunity to dissociate between bottom-up feedforward (FF) and modulatory feedback (FB) responses which were activated in separated layers of a cortical unit. Among the fMRI methods, blood oxygen level dependent (BOLD) contrast has been the gold standard for nearly three decades (P. A. Bandettini et al. 1992; Ogawa et al. 1990) due to its ability in rapid acquisition using Gradient Echo (GE)–based echo planar imaging (EPI) (P. Mansfield 1977). However, the draining-vein effect poses a significant challenge to layer-dependent fMRI using BOLD contrast. This is primarily because BOLD contrast is T2*-based and sensitive to changes in magnetic field inhomogeneity caused by varying concentrations of deoxygenated hemoglobin. While arteries, capillaries, and draining veins all contribute to BOLD responses, the draining veins are considered the major contributor because of the larger volume and lower baseline oxygenation level compared to arteries (Boas et al. 2008). The intracortical veins run perpendicular to the cortical surface and drain blood into pial veins. Consequently, BOLD responses induced in lower cortical layers will be carried to superficial layers, the so-called ‘leakage model’ (Markuerkiaga, Barth, and Norris 2016).

To address the draining-vein issue in layer BOLD fMRI, one approach is to estimate depth-dependent BOLD signal by the inverse calculation of leakage model (Havlicek and Uludag 2020; Heinzle et al. 2016). However, the model-based vein removal methods are primarily exploratory and the effectiveness is still in need of validation (L. R. Huber et al. 2021). Besides, the phase information of BOLD fMRI has also been used to reduce the draining-vein contamination. The BOLD signal arising from oriented vessels such as ascending and pial veins results in a coherent phase shift, while the BOLD signal from capillaries tends to have near-zero net phase (Menon 2002). Therefore, the macrovascular signal variation may be captured by time-dependent phase variation and could be removed through phase regression. A recent study employed this phase regression technique to investigate laminar activity profiles and demonstrated that the phase-regressed results from GE-EPI are comparable to those from Spin Echo (SE) EPI, which is considered less susceptible to draining-vein contamination (Stanley et al. 2021). Nevertheless, SE-EPI is not completely free from T2* effects either due to the long readout time (Goense and Logothetis 2008) and, therefore, is not completely free from draining-vein effects. In another layer-dependent fMRI study focused on a finger-tapping task, phase regression significantly reduced the bias toward the superficial layer in the primary motor area (M1), although some residual macrovascular contribution remained (Knudsen et al. 2023).

Apart from the BOLD contrast, vascular space occupancy (VASO) fMRI, a non-BOLD method, has been gaining popularity in recent years (L. Huber et al. 2021a). VASO fMRI measures the changes of cerebral blood volume (CBV) rather than the level of blood oxygenation. These CBV changes primarily originate from small arterioles located close to layer-dependent neural activation sites (Drew, Shih, and Kleinfeld 2011; Gagnon et al. 2015). In contrast, the larger vessels in the downstream are expected to have limited contribution on the overall CBV changes (Drew, Shih, and Kleinfeld 2011; Gagnon et al. 2015; O’Herron et al. 2016; Takano et al. 2006). Although VASO imaging demonstrates high spatial specificity, the spatial coverage of layer-fMRI VASO acquisition is limited due to the nature of the contrast-generation mechanism (L. Huber et al. 2018; L. Huber, Uludağ, and Möller 2019). Even with the advanced version of VASO, namely Multiple Acquisitions with Global Excitation Cycling (MAGEC) VASO, the acquisition time for a 83.2-mm-thick volume is 8.2 s (L. Huber et al. 2021b). Additionally, it is challenging for VASO sequences to measure CBV in subcortical structures such as the lateral geniculate nucleus (LGN) because the arterial arrival time is too short for reproducible measurements (https://layerfmri.com/2021/02/22/vaso_ve/#more-2688). Even in the only VASO study that succeeded in measuring hippocampal CBV, the experiment required multiple inversion pulses, and the spatial resolution was relatively low (1×1×4 mm3) compared to that of layer-fMRI VASO (Rane et al. 2016).

In addition to layer-fMRI VASO method, several magnetization preparation sequences with turbo fast low angle shot (TurboFLASH) readout (Matthaei et al. 1985) have been proposed to improve spatial specificity. These include T1*ρ* preparation, T2 preparation and diffusion-weighted T2 preparation. TurboFLASH readouts are used instead of EPI readouts to minimize T2* contamination. In the T1*ρ* preparation sequence, the spin-lock editing pulses were used to enhance the contribution from microvasculature and reduce the influence of large veins (Rane et al. 2014). In the case of the T2 preparation sequence, the intravascular contribution to the BOLD signal can be reduced at high magnetic fields such as 7T (Hua et al. 2014). Nevertheless, the detection sensitivity and spatial specificity of T1*ρ* and T2 preparation sequences were shown to be lower than a VASO sequence empirically (L. Huber, Hua, et al. 2017; L. Huber et al. 2021a). To further enhance spatial specificity, small diffusion-weighted crusher gradients are included in the T2 preparation sequence. These small diffusion-weighted gradients, also referred to as velocity-nulling (VN) gradients, suppress signals from moving blood. Although the influence of large veins and arteries is reduced by the velocity-nulling gradients, the sensitivity of the diffusion-weighted T2 preparation sequence is likely compromised due to incomplete refocusing (L. Huber, Hua, et al. 2017).

To improve the detection sensitivity of sub-millimeter fMRI sequences with VN gradients, one possibility is to incorporate VN gradients into GE-EPI sequences. The sensitivity of GE-BOLD acquisition is 20–30% higher than SE-BOLD acquisition (Peter A. Bandettini et al. 1994; Yacoub et al. 2005). However, the origins of the GE-BOLD signal are dominated by large vessels. By applying the VN gradients, the contribution from pial and draining veins could be substantially suppressed. Using GE-BOLD sequences also circumvents the issue of incomplete refocusing found in diffusion-weighted T2 preparation sequences. Nonetheless, the VN gradients, which were inserted between the RF pulse and the EPI readout, prolonged the echo time (TE), imposing the difficulty in maintaining the TE around the T2* value. This is especially problematic for sub-millimeter fMRI at 7T because the optimal TE is too short to accommodate the VN gradients.

This current study investigated the depth-dependent spatial specificity of sub-millimeter fMRI with VN gradients at 3T. The optimal echo time (TE) is in the range of 40–50 ms, matching the T2* value of grey matter at 3T, which is sufficiently long to incorporate VN gradients. To offset the reduction in the signal-to-noise ratio (SNR) when transitioning from 7T to 3T, the NOise Reduction with DIstribution Corrected principal component analysis (NORDIC PCA) was employed (Vizioli et al. 2021). The application of the NORDIC denoising method has been empirically shown to enhance the SNR by more than two-fold.

In this study, we initially implemented fMRI with a 0.9 mm isotropic resolution and VN gradients at 3T. We then examined the depth-dependent spatial specificity within the primary motor area (M1) using a finger-tapping experiment (L. Huber, Handwerker, et al. 2017). During the stage of optimizing the b value of the VN gradients, we restricted our acquisition to a slab encompassing M1 to avoid potential confounding from image reconstruction under a high acceleration rate. After determining a suitable b value, we employed the Simultaneous Multislice (SMS) method (Setsompop et al. 2012) for volumetric acquisition. Ultimately, we achieved the brain-wide acquisition with 0.9-mm isotropic resolution, 111.6 mm of field-of-view (FOV) thickness and repetition time (TR) of 3.84 s, thereby validating the depth-dependent spatial specificity. Utilizing a visual checkerboard experiment, we further demonstrated that the VN gradient led to only a marginal reduction in BOLD sensitivity at the lateral geniculate nucleus (LGN) – an area typically more challenging to measure compared to cortical regions. Building on the demonstrated BOLD sensitivity and spatial specificity, we explored the feasibility of conducting brain-wide directed functional connectivity studies at 3T. The results showcased several instances of directed functional connectivity that aligned with previous reports in the literature.

## Theory

### Draining-vein suppression using velocity-nulling gradient

**Figure 1:**
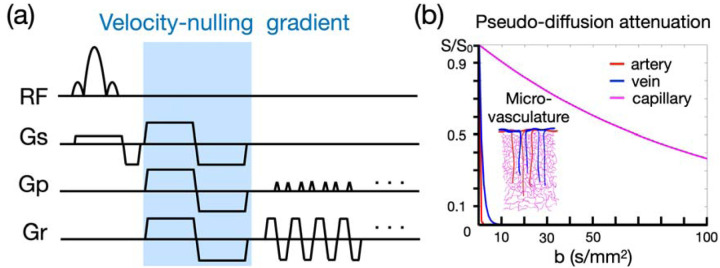
Velocity-nulling (VN) gradient in GE-EPI. (a) The diagram of pulse sequence. The VN gradient is highlighted in light blue. (b) The simulated signal attenuation against b values.

The BOLD measurement at cortical layers is spatially blurred and biased towards superficial layers due to the draining-vein effect (Turner 2002; Duong et al. 2003). Here we incorporate a velocity-nulling (VN) gradient into GE-EPI to supress the signal from draining veins (see Figure 1a). The signal attenuation against the b value will generally follow the exponential decay in the Intravoxel Incoherent Motion (IVIM) process (Le Bihan 2019). The IVIM process models the collective motion of water molecules in blood within a vessel network as they transition from one vessel segment to another. This collective movement can be perceived as a pseudo-diffusion process where average displacements equate to the mean vessel segment length and the mean velocity matches that of the blood in the vessels.

In a capillary network, the average displacement is approximately 100 μm and the mean velocity is around 1 mm/s, yielding a pseudo-diffusion coefficient (D*) of 10^−8^ m^2^/s. In the case of cortical penetrating veins, the actual flow velocity is difficult to measure due to their small size. However, optical imaging suggests a mean flow velocity of ~2.5 mm/s in these veins (Shih et al. 2013). Given the assumption that blood primarily flows along the penetrating veins, the average displacement is set as 3 mm, typically matching the cortical thickness of the human cortex. Accordingly, the pseudo-diffusion coefficient for penetrating veins is ~7.5×10^−7^ m^2^/s. In contrast , the flow velocity in cortical arteries is ~12 mm/s (Schaffer et al. 2006), with an average displacement also of 3 mm. This configuration leads to a pseudo-diffusion coefficient of ~3.6×10^−6^ m^2^/s.

The signal amplitude that underlies the effect of the VN gradient follows an exponential decay as represented by the equation S/S_0_ = exp [–b (D* + D_blood_)]. In this formula, S denotes the vascular signal affected by the VN gradient, S_0_ denotes the signal strength without the VN gradient, b denotes the b value of diffusion gradient, D* indicates the pseudo diffusion coefficient, and D_blood_ indicates the water diffusion coefficient in blood. As the D* ≫ D_blood_, the signal decay equation can be simplified to S/S_0_ = exp (–b•D*). Using the predetermined pseudo diffusion coefficients for arteries, veins, and capillaries, we simulated the signal attenuations as illustrated in Figure 1b. It is important to highlight that the b value represented in Figure 1b pertains to a single axis. Our results imply that a relatively small b value should effectively suppress the contribution from draining veins, yet simultaneously cause only minimal attenuation of signals originating from capillaries. Taking into account the varying orientation of penetrating veins within the highly convoluted human cortex, an equivalent degree of diffusion weighting is applied across all axes, which triples the ultimate b value relative to that of a single axis. For example, using a b value of 30, equivalent to a single-axis b value of 10, can suppress the signal from veins to less than 0.1%, albeit with a consequential loss of 26% of the signal from capillaries.

## Results

### The efficacy of draining-vein suppression using velocity-nulling (VN) gradient

**Figure 2:**
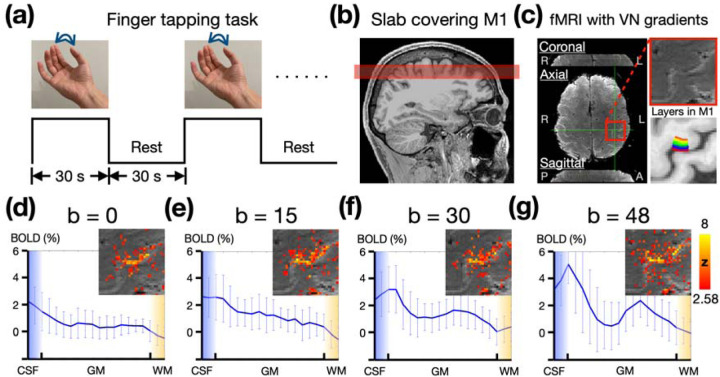
Empirical results of finger tapping task with different strength of draining-vein suppression from a single participant. (a) The paradigm of finger tapping task. (b) The illustration of the slab acquisition covering M1. (c) The slab image with 0.9-mm isotropic resolution in three orthogonal views. The 20 layers of M1 are color-coded as shown in the bottom-right panel. (d-g) The depth-dependent profiles of BOLD activation at M1 associated with b=0, 15, 30, 48. The statistical maps were corrected (uncorrected p < 0.01; corrected p < 0.05) and color-coded as indicated by the color bar.

We evaluated the efficacy of draining-vein suppression by studying the depth-dependent profile of BOLD activation at M1, utilizing the finger-tapping task illustrated in Figure 2a. We acquired slabs encompassing M1 with a range of b values of the VN gradient, as demonstrated in Figure 2b. The left panel in Figure 2c presents the denoised slab image in three orthogonal views. The area around M1 was highlighted within a red box, and a zoom-in view of this region was shown in the upper-right panel. A color-coded layer representation within M1, overlaid on the T1 image, is shown in the bottom-right of Figure 2c. The depth-dependent profiles of BOLD activation at M1 were extracted using the voxel-based approach. The depth-dependent profiles corresponding to a various of b values are shown in Figure 2d–g. Overall, the results suggest that the VN gradient efficiently mitigated the draining-vein effect, thus achieving the desired laminar specificity at 3T. Both b values of 30 and 48 exhibited double-peak response patterns, yet the spatial variance in the profile with b = 30 was lower. Moreover, employing a b value of 48 may have overly suppressed the signal from capillaries, as simulated in Figure 1b. Therefore, we utilized a b value of 30 for the VN gradient in the subsequent experiments.

### The impact of SMS acceleration rate on depth-dependent profiles

**Figure 3:**
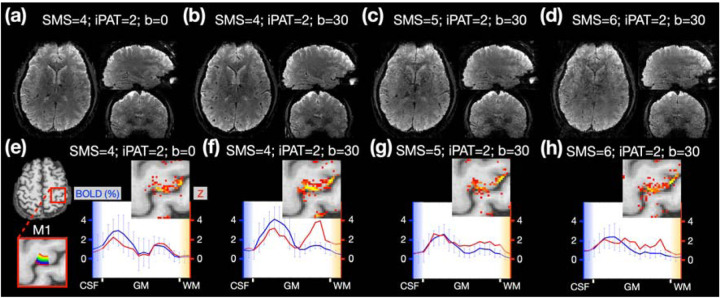
Sub-mm fMRI with brain-wide coverage in one participant. (a-d) The reconstructed images with different combination of SMS factor and b value. (e-h) The activation maps and depth-dependent profiles associated with different scanning protocols. The number of volumes acquired during the motor task was maintained at 282 across all protocols.

To expand spatial coverage from a slab to a volume, both in-plane and SMS acceleration are required. The combined acceleration rate is defined as the product of in-plane and SMS accelerations. As higher acceleration rates may adversely affect SNR and signal quality, it is crucial to understand their impact on signal quality and the depth-dependent profile. Our tests on imaging protocols employed SMS factors of 4, 5, and 6 in combination with a VN gradient of b = 30. Additionally, we examined the protocol using an SMS factor of 4, but without a VN gradient. Figure 3a–Figure 3d showcase reconstructed images with SMS factors ranging from 4 to 6. The TRs corresponding to SMS factors of 4, 5, and 6 are 3.84s, 3.08s, and 2.58s, respectively, with an isotropic spatial resolution of 0.9 mm maintained throughout. The optimal image quality was realized with an SMS factor of 4. As the SMS factor was elevated from 5 to 6, minor image artifacts started to appear, particularly in the anterior subcortical and anterior frontal regions, as shown in Figure 3c and Figure 3d. Similarly, the depth-dependent profiles of M1 activation in Figure 3e and Figure 3f display a double-peak pattern with an SMS factor of 4. This pattern demonstrated greater spatial stability with the application of the VN gradient, as indicated by increased statistical significance in the deeper layers (refer to Figure 3f). The depth-dependent profiles depicted in Figure 3g and Figure 3h showed increased spatial instability, likely attributable to feature loss when the combined acceleration rate was too high.

### The impact of TE on depth-dependent profiles

**Figure 4:**
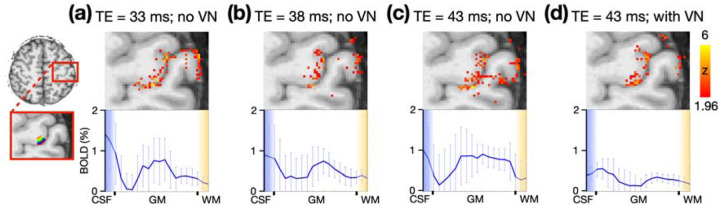
Illustration of TE impact on activation maps and depth-dependent profiles. A total of four finger-tapping experimental sessions were conducted on a single participant with varying TEs: (a) 33 ms, (b) 38 ms, (c) 43 ms, and (d) 43 ms with the VN gradient. No VN gradient was applied in (a)-(c). The red rectangle in the left-most column highlighted an enlarged view centered on the M1 region. Within M1, a total of 20 layers are represented and color-coded in the bottom-left panel. The statistical maps were corrected (uncorrected p < 0.05; corrected p < 0.05) and color-coded as indicated by the color bar on the far right. The images are presented in radiology view. The bottom row presents the enlarged views of the depth-dependent profiles, while the whiskers indicate the standard deviation across voxels within each individual layer.

To understand the impact of TE on the depth-dependent profiles within M1, a single participant completed four finger-tapping sessions with varied TEs. The resulting activation maps and depth-dependent profiles are displayed in Figure 4. The TEs in conventional fMRI are 33, 38, and 43 ms are shown in Figure 4a, Figure 4b, and Figure 4c, respectively, all without the application of the VN gradient. Those maps and profiles exhibited a notable bias towards the cerebral spinal fluid (CSF) located exterior to the cortical surface, with none displaying the double-peak patterns. In contrast, the depth-dependent profile with the VN gradient, as shown in Figure 4d, displayed reduced bias towards CSF and exhibited the typical double-peak pattern. These results underscored the indispensable role of the VN gradient in draining-vein suppression.

### The effectiveness of phase regression method for macrovascular suppression

**Figure 5:**
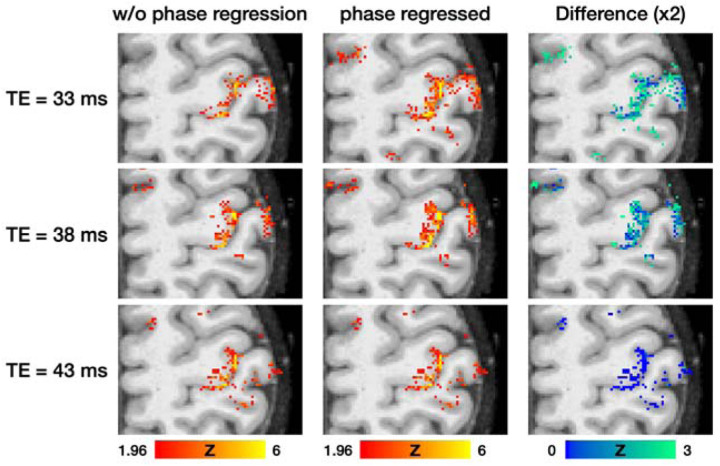
Influence of TE and phase regression on BOLD activation maps in a finger-tapping task. The rows represent the maps associated with TEs of 33 ms, 38 ms, and 43 ms, from top to bottom, respectively. Columns from left to right display the non-phase-regressed, phase-regressed, and difference maps, respectively. The right-most column depicts the absolute difference between the non-phase-regressed and phase-regressed maps. The activation maps were corrected statistically (uncorrected p < 0.05; corrected p < 0.05). The z values in each column are color-coded as indicated by the color bars.

To evaluate the impact of phase regression on BOLD activation maps as a function of TE, we applied phase regression to the fMRI datasets that were used in Figure 4, excluding the dataset with the VN gradient. In Figure 5, the left and middle columns represent BOLD activation maps without and with phase regression, respectively, while the right column displays the absolute difference between them. The rows correspond to TEs of 33 ms, 38 ms, and 43 ms, from top to bottom. The difference maps demonstrate that the impact of phase regression is most prominent at a TE of 33 ms, diminishing slightly as TE increases to 38 ms, and becoming minimal at 43 ms. Given that macrovascular contributions to the BOLD signal at 3T decrease with increasing TE (Irati Markuerkiaga et al. 2021), this reduced efficacy of phase regression along TE may be attributed to a diminished influence from large vessels. Moreover, at a TE of 43 ms, Figure 5 indicates that phase regression has a marginal effect whereas Figure 4 suggests that the VN gradient remains effective. This suggests that the impact of phase regression on smaller vessels, such as penetrating veins, may be limited.

### BOLD sensitivity in subcortical regions

**Figure 6:**
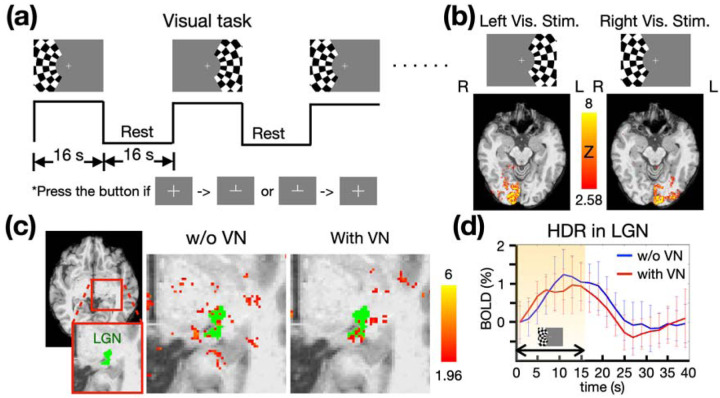
The visual task paradigm and the corresponding results from a single participant. (a) The checkerboard flashed alternately on the left and right sides for 16 seconds, followed by a 16-second resting period. (b) The BOLD activation maps correspond to the visual stimuli in the left or right visual hemifield. (c) The BOLD activation maps in the LGN, presented with and without a VN gradient. The z-statistics are overlaid on the individual T1 image and color-coded as indicated by the color bar. (d) The HDRs in the LGN, compared without and with a VN gradient (b value of the VN gradient is 30). The shaded area highlights the period of visual stimulation. The blue and red traces denote the mean HDRs of BOLD signal change obtained using the protocols without and with VN gradient, respectively. The whiskers denote the standard deviation across trials.

To analyze how BOLD sensitivity is influenced by the VN gradient, we applied a visual stimulus in the form of a checkerboard presentation, depicted in Figure 6a, to investigate the subcortical BOLD response in the LGN. As illustrated in the left panel of Figure 6b, the visual cortex elicited significant BOLD responses in the right hemisphere when the visual stimuli were introduced in the left visual hemifield. Similarly, stimuli in the right visual hemifield elicited BOLD responses in the left hemisphere. Figure 6c highlights the location of the LGN in green and displays the activation maps both without and with the VN gradient. To evaluate the impact of the VN gradient on the hemodynamic responses (HDRs) in the LGN, we averaged the corresponding event-related responses within the significantly-activated LGN voxels, and then performed an average across trials. The HDRs in the LGN, both without and with the VN gradient, are presented in Figure 6d. The time courses were re-sampled with a TR of 2s using linear interpolation for better visualization. Although the averaged HDR without the VN gradient demonstrated a slightly stronger BOLD response compared to the HDR with the VN gradient, this difference was marginal and exhibited no statistical significance. These findings suggest that the VN gradient does not significantly attenuate the activation strength within the LGN.

### Robustness of depth-dependent activation profile

**Figure 7:**
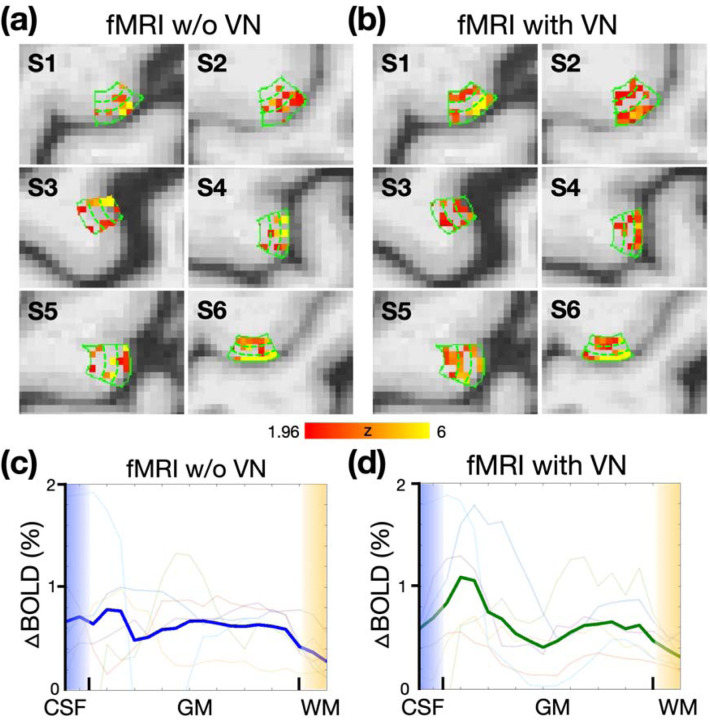
Activation maps and depth-dependent profiles of BOLD responses across participants. (a) BOLD activation maps in the absence of a VN gradient, with the solid green contour delineating the primary motor cortex (M1). Dashed traces delineate superficial, middle, and deep cortical layers. (b) Activation maps with a VN gradient applied. (c) Depth-dependent profiles of BOLD response in the absence of a VN. The dark blue traces denote the group-averaged data. (d) Depth-dependent profiles with VN applied. The dark red traces denote the group-averaged profiles with the VN gradient applied. In both (c) and (d), the traces with light colors indicate individual profiles.

From Figure 2 to Figure 4, the double-peak patterns were successfully demonstrated for a single participant. However, the consistency of this depth-dependent profile across a broader participant base has yet to be confirmed. Activation maps for six participants, both without and with the VN gradient, are shown in Figure 7a and Figure 7b, respectively. While one participant from the group without the VN gradient displayed the double-peak pattern, the majority of the depth-dependent profiles appeared spatially unstable. Consequently, the group-averaged depth-dependent profile failed to prominently exhibit the double-peak pattern. In contrast, in the VN fMRI data, the middle layers generally showed reduced activation. Hence, the double-peak pattern was evident in the group-averaged depth-dependent profile, as depicted in Figure 7d.

### Brain-wide directed functional connectivity

**Figure 8:**
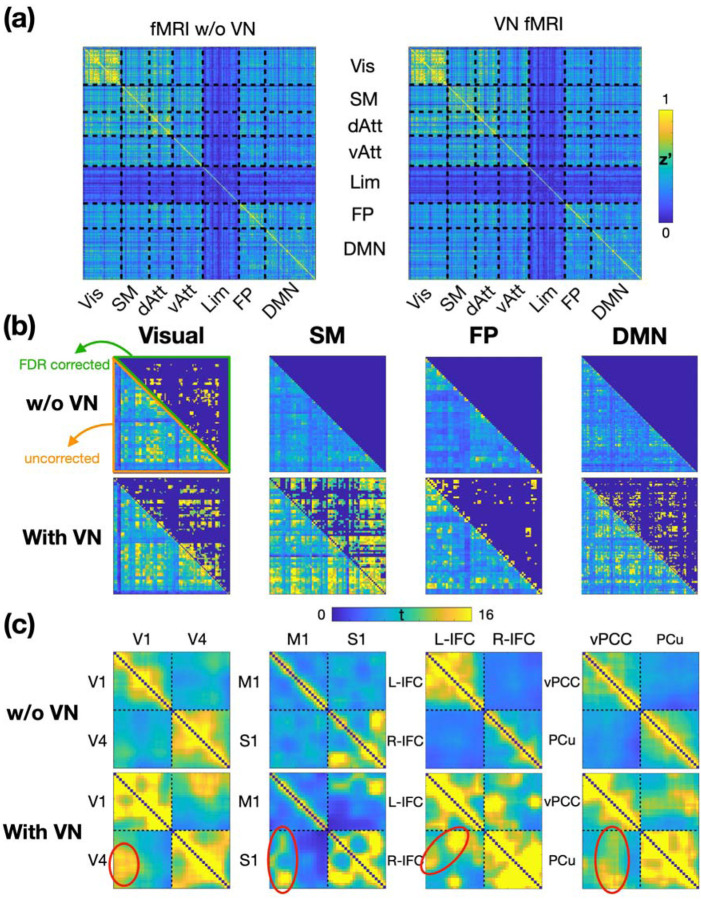
Directed functional connectivity across functional networks (N = 6). (a) Fisher’s z transformed FC matrices. The FC matrix on the left represents the Fisher’s z values without a VN gradient, while the matrix on the right corresponds to the Fisher’s z values with a VN gradient. (b) Statistical results of FC within the visual, sensorimotor, frontoparietal, and default-mode networks. The matrix’s lower triangular parts show uncorrected t-values, while the upper triangular parts show t-values that survived FDR correction (corrected p < 0.001). (c) Depth-dependent FC matrices for a representative ROI pair in each network. T-values are color-coded as indicated by the color bar. Red circles highlight directed functional connectivities of particular interest. The upper row displays the results without a VN gradient, and the lower row presents the results with a VN gradient. Abbreviations: z’ – Fisher’s z; M1 – primary motor cortex; S1 – primary sensory cortex; L-IFC – left inferior frontal cortex; R-IFC – right inferior frontal cortex; vPCC – ventral posterior cingulate cortex; PCu – precuneus; Vis – visual network; SM – sensorimotor network; dAtt – dorsal attention network; vAtt – ventral attention network; Lim – limbic network; FP – frontoparietal network; DMN – default-mode network.

The group-averaged Fisher’s z-transformed FC matrices are presented in Figure 8a, with ROIs organized according to their respective functional networks. The FC matrix on the left was derived from conventional fMRI data without the application of VN gradients, while the matrix on the right was generated using VN fMRI data. The group mean of Fisher’s z values exhibited comparable outcomes between conventional fMRI and VN fMRI datasets. In Figure 8b, the group-level statistics for the directed functional connectivity matrix are presented, including the Visual (Vis), Sensorimotor (SM), Frontoparietal (FP), and Default-Mode Networks (DMN). The lower triangular part of the matrix displays uncorrected t-values, as derived from independent t-tests. The upper triangular part shows only the t-values that survived the False Discovery Rate (FDR) correction procedure (corrected *p* < 0.001) (Benjamini and Hochberg 1995; Benjamini and Yekutieli 2001). In the absence of a VN gradient, scarcely any of the FCs remained statistically significant following FDR correction, with minor exceptions within the visual network. Conversely, when VN fMRI is utilized, all examined functional networks exhibited statistically significant FCs. These findings suggest that VN fMRI enhances statistical power by effectively reducing inter-participant variability.

The layer specificity also enables the directed brain connectome as demonstrated in Figure 8c. Examples from each functional network are presented, specifically focusing on those ROI pairs that survived FDR correction in the VN fMRI dataset. Directed FCs of interest are denoted by red circles. Within the visual network, the primary visual cortex (V1) exhibited feedforward connectivity originating from its superficial layers and targeting the middle layers of the visual area V4. In the sensorimotor network, the M1 received inputs exclusively in its superficial layers, which originated from both the superficial and deep layers of the primary sensory cortex (S1). In the frontoparietal network, a reciprocal feed-forward relationship was observed between the left inferior frontal cortex (L-IFC) and the right inferior frontal cortex (R-IFC), originating predominantly from superficial to middle layers. In the default mode network (DMN), the posterior cingulate cortex (PCC) exhibited connectivity dominated by middle layers. The precuneus (PCu) also showed feedforward connectivity from PCC’s superficial to its own middle layers, which is consistent with its role as a major hub in the DMN.

## Discussion

To develop brain-wide directed functional connectivity using depth-dependent functional information, several technical criteria must be met: sub-mm spatial resolution, high spatial specificity, adequate temporal resolution, sufficient functional sensitivity, and global brain coverage. In this study, we addressed those challenges by incorporating a number of techniques, such as velocity-nulling gradients, SMS acceleration and NORDIC denoising techniques. As a result, we were able to develop a VN fMRI sequence featuring 0.9-mm isotropic spatial resolution, a TR of 3.84 seconds and brain-wide coverage. The effects of various parameters, including the TE and the b value associated with the VN gradient, were systematically evaluated. Our VN fMRI showcased its spatial specificity by revealing double-peak patterns within M1 during a finger-tapping task. Compared to conventional fMRI approaches, the VN fMRI exhibited greater robustness across participants. Furthermore, by properly selecting the b value of the VN gradient based on empirical evidence, our VN fMRI was capable of detecting BOLD signals not only at the cortical surface but also in subcortical regions, specifically the LGN. This optimized imaging framework enables the detailed examination of directed functional connectivity, demonstrating several layer-specific functional relationships consistent with the existing literature.

In 7T layer-dependent fMRI, bipolar diffusion gradients have been employed to mitigate the draining-vein effect, but in conjunction with T2 preparation methods (L. Huber, Hua, et al. 2017) rather rapid GE-EPI sequences. This is likely due to the limited TE associated with GE-EPI at 7T, which is insufficient to accommodate bipolar diffusion gradients in an ultra-high-resolution regime. However, it is noteworthy that the functional sensitivity of diffusion-weighted T2 preparation methods is considerably lower than the GE-BOLD method (L. Huber et al. 2021a). While researchers have explored higher magnetic field strengths to enhance sensitivity, the current study introduces a depth-dependent GE-EPI approach implemented at 3T. While this may initially appear counterintuitive, the application of GE-EPI at 3T affords several unique advantages. First, the extended T2* values at 3T lead to diminished T2* blurring along the phase-encoding direction, thus yielding a more focused point-spread function (PSF). Second, geometric distortions and signal losses due to susceptibility effects are less prominent at 3T relative to those at 7T. Third, the feasibility of conducting layer-dependent fMRI at 3T broadens the scope for multi-site, large-scale investigations. These attributes are of critical importance for a broad range of neuroscience applications.

To date, only a limited number of studies have reported on 3T layer-specific GE-BOLD fMRI (Koopmans, Barth, and Norris 2010; Scheeringa et al. 2016; Irati Markuerkiaga et al. 2021; Knudsen et al. 2023). Among these, the study by Knudsen et al. (2023) addressed the draining-vein contamination issue by employing phase regression techniques to mitigate bias toward superficial cortical layers. Phase regression operates by removing signals from vessels that generate a non-zero net phase, typically vessels that are large and oriented in a particular direction. As illustrated in Figure 5, the efficacy of phase regression diminishes slightly at a TE of 38 ms and becomes almost negligible at a TE of 43 ms. This reduction in efficacy suggests a minimal contribution from macrovascular sources to the BOLD signal at extended TEs. In contrast, Figure 4d demonstrates that VN gradients are still effective even at a long TE, likely attributable to the suppression of smaller, cortically-penetrating veins. Our findings suggest that while a phase regression method minimizes the macrovascular contribution to BOLD signal, VN gradients further suppress the signals from smaller, cortically-penetrating veins.

Although the VN gradients suppress the intravascular signal, the gradient itself does not address the extravascular effect that may influence the BOLD signals in middle and deep cortical layers, potentially causing vascular blurring across cortical layers. However, the extended TE due to the insertion of a VN gradient may alleviate this issue. Our results in Figure 5 suggest that extended TE has an impact similar to that of the phase regression approach which has been shown to reduce both intravascular and extravascular contributions from pial veins (Stanley et al. 2021). The extent to which the extravascular contribution is reduced warrants further investigation. Notably, in some double-peak activation patterns observed in this study, the BOLD amplitude corresponding to superficial layers substantially exceeded that related to deeper layers. This discrepancy, not evident in electrophysiological data (Maier 2010), suggests potential residual extravascular effects. Therefore, caution is advised when interpreting BOLD amplitude as a direct indicator of neural activity strength. This consideration is less pertinent in layer-specific FC analyses due to the normalization of amplitude in the computation of correlation coefficients.

Beyond the use of GE-BOLD fMRI at a 3T magnetic field strength, the MAGEC VASO sequence has been shown to achieve both spatial specificity and volumetric brain coverage (~83 mm thickness), albeit with a relatively long TR of 8.2 seconds. Importantly, the energy spectrum of resting-state signals generally falls within 0.01 to 0.1 Hz range. According to the Nyquist sampling theorem, a TR shorter than 5 seconds is required to avoid temporal aliasing. Exceeding this limit can lead to frequency aliasing, wherein signals at the higher end of the bandwidth overlap with signals in other bands, thereby complicating the interpretation of resting-state FC. Our VN fMRI approach, featuring a TR of less than 4 seconds, effectively alleviates this issue of temporal aliasing.

Leveraging the ultrahigh-resolution, brain-wide coverage and layer specificity provided by the proposed VN fMRI, our results of directed FC in Figure 8c exhibit several established feedforward and feedback neural pathways. Within the visual network, our results demonstrate feedforward signal propagation from the superficial neural layers of the primary visual cortex (V1) to the intermediate layers in the visual area 4 (V4). This feedforward connection is a component in the ventral stream of visual pathways (Goodale and Milner 1992; Kravitz et al. 2013). Additionally, within the sensorimotor network, we observed directed FC originating from the primary somatosensory cortex (S1) and targeting the superficial neural layers of M1. The FC of the posterior cingulate cortex (PCC) was predominantly observed in the middle laminae. These observations are in alignment with previously reported findings from a laminar-specific fMRI study (L. Huber, Handwerker, et al. 2017).

Despite the unprecedented features of our VN fMRI, this study is not without limitations, outlined as follows. 1) Residual intravascular signal: the VN gradients suppress signals originating from vascular flow oriented in specific directions. However, when vasomotion lacks coherence, the effective pseudo-diffusion coefficient (D*) is reduced, thereby diminishing the efficiency of intravascular signal suppression. In Figure 3, Figure 4 and Figure 5, residual intravascular signals are predominantly localized around regions of the pial surface exhibiting elevated curvature. This is likely attributable to incoherent flow at the branching junctures of pial vessels. Notably, these residual intravascular signals are generally confined to the vascular space, thus exerting minimal impact on the depth-dependent signal characteristics within the grey matter. 2) Inadequate TR for event-related paradigms: Although our VN fMRI acquisition reduced the TR from 8.2 s to 3.84 s, this TR duration remains suboptimal for studies employing event-related task designs. Future research would benefit from enhanced acceleration capabilities to facilitate the investigation of high-level cognitive processes. Such developments, however, are beyond the scope of this study.

## Conclusion

In summary, the developed VN fMRI exhibited layer specificity and BOLD sensitivity successfully at 3T. Leveraging its brain-wide coverage and reasonable scan time, the VN fMRI yielded promising results in the study of directed FC. Given the widespread accessibility of 3T scanners, the potential impact of this development is expected to be extensive across various domains of neuroscience research.

## Materials and Methods

### Participants

In this study, we recruited a total of six healthy adults (2 females, age = 32.3 ± 8.5 years). All participants underwent screening to confirm no history of neurological or psychiatric conditions, previous head trauma, or MRI contraindications. Only right-handed individuals are included in this study. Prior to participation, each individual provided informed consent in accordance with the experimental protocol approved by the University of North Carolina at Chapel Hill Institutional Review Board.

### Stimulation paradigms

All visual stimuli across the paradigms were presented using PsychoPy software (Version 2022.2.4) (Brainard 1997). The stimuli were displayed on a screen positioned at the head end of the magnet bore, and participants viewed the visual presentations through a mirror mounted on the head coil.

#### Finger-tapping task

In a finger tapping task, the primary motor cortex (M1) receives incoming sensory and associative information in its superficial layers. This activation subsequently propagates to the deeper layers, where output signals are generated to ultimately control finger muscles. Consequently, both the superficial and deep layers of M1 exhibit activation, while the middle layers typically do not. Such a double-peak response pattern at M1 has been used as a hallmark to evaluate the spatial specificity in layer-dependent fMRI studies (L. Huber, Handwerker, et al. 2017).

During the experiment, participants were instructed to perform a pinch-like tapping action between the thumb and index finger of their right hand. The timing and frequency of the finger tapping was synchronized with a video displayed on a screen within the scanner. The video consisted of a block-designed paradigm, as illustrated in Figure 2a. The tapping frequency was 2 Hz with each ‘ON’ block spanning 30 seconds, immediately followed by a 30-second rest during the ‘OFF’ block. Depending on the repetition time (TR), the total number of blocks varied between 12 and 18. The overall numbers of fMRI volumes in finger-tapping tasks were maintained at either 282 or 286.

#### Visual task

The design of visual experiment is illustrated in Figure 6a. The visual stimulus consisted of a high-contrast visual checkerboard, with its contrast reversing at a frequency of 8 Hz. The checkerboard alternately flashed on the left or right for 16 seconds, followed by a 16-second rest period. Participants were instructed to press a button on a response box when the crosshair changed from ‘+’ to ‘⊥’ or from ‘⊥’ to ‘+’. These responses were recorded to verify the compliance to the instructions.

### Common protocol in MR acquisitions

MR images were acquired using a Siemens 3T Prisma scanner (Siemens Healthcare, Erlangen, Germany) and a 32-channel head coil at the Biomedical Research Imaging Center (BRIC) at the University of North Carolina at Chapel Hill. For each participant, an MPRAGE image was acquired for structural imaging using the following imaging parameters: 0.8-mm isotropic resolution, TR/TE/TI = 2400/2.24/1060 ms, flip angle = 8°, in-plane acceleration factor = 2, partition thickness = 0.8 mm, 208 partitions, sagittal slicing, image matrix = 320 × 300, and FOV = 25.6 cm × 24.0 cm.

For functional imaging, the customized SMS sequence with VN gradient was implemented in the vendor-provided IDEA environment (VE11E). The functional data were acquired using a blipped-controlled aliasing in parallel imaging (blipped-CAIPI) SMS imaging (Setsompop et al. 2012) with two-dimensional (2D) single-shot EPI readout. While imaging protocols varied slightly across different experiments, they all shared the following parameters: isotropic spatial resolution of 0.9 mm; axial slicing; in-plane image dimensions of 224×210; frequency and phase encoding in the left-right and anterior-posterior directions, respectively; and an in-plane acceleration rate of 2. A 50-mm-thick saturation band was utilized to suppress the signal from the eyeballs. Additionally, a brief fMRI acquisition with an opposite phase-encoding direction was acquired for distortion correction.

### Experiment configuration and imaging protocols in optimization phase

The data acquisitions in this study consisted of two phases: optimization and validation. During the optimization phase, various scan protocols were evaluated to establish the optimal scan parameters. To minimize inter-subject variation, data were solely collected from a single participant. This phase included two experiments: 1) b-value optimization and 2) acceleration rate optimization.

#### Experiment 1A: Optimization of b value in VN gradient

Empirical tests were conducted with b values of 0, 15, 30 and 48, based on the simulation results shown in Figure 1b. A finger-tapping task was employed to assess BOLD sensitivity and spatial specificity. To avoid confounds from reconstruction errors associated with a high acceleration rate at this preliminary stage, we acquired a slab encompassing the primary motor cortex (M1) without SMS acceleration, as shown in Figure 2b. The slab placement was under the supervision of an on-site radiologist. With the acquisition of 21 slices in total, the resultant slab thickness was 18.9 mm. The TEs corresponding to these b values were 33, 41, 43, and 45 ms, respectively, while the TR was consistently maintained at 2.54 s. The total number of acquired volumes was 286. The total number of finger-tapping blocks was 12.

#### Experiment 1B: Optimization of SMS acceleration rate

Higher SMS acceleration rates can decrease the volumetric scan time of fMRI, which potentially enhances its statistical power and captures a broader range of temporal dynamics. However, a high acceleration rate might also compromise the quality of image reconstruction (Cauley et al. 2014; Todd et al. 2016). The relationship between SMS acceleration and laminar activation profiles is still unclear. In contrast, the volumetric scan time of fMRI should not be excessively prolonged. Given that the majority of energy in the BOLD signal spectrum falls between 0.01 and 0.1Hz, it is preferable to ensure that the TR does not exceed 4 seconds. Hence, we tested SMS acceleration rates of 4, 5, and 6 with an in-plane acceleration rate of 2, resulting in TRs of 3.84, 3.11, and 2.63 seconds, respectively. The finger-tapping task was employed to assess the impacts on laminar activation profiles.

We conducted four tests using different scanning parameters: 1) a SMS factor of 4 without VN gradient, 2) a SMS factor of 4 with VN gradient, 3) a SMS factor of 5 with VN gradient, and 4) a SMS factor of 6 with VN gradient. The b value of VN gradient was set as 30 based on preliminary results from Experiment 1A. The TE and total number of acquired volumes were kept constant at 43 ms and 282 ,respectively. The numbers of slices per test were 124, 124, 125, and 126, respectively, and the total number of finger-tapping blocks per test were 12, 12, 14, and 18, respectively. The spatial shifts caused by CAIPI blips for SMS factor of 4, 5, 6 were FOV/2, FOV/3 and FOV/3 respectively.

### Experiment configuration and imaging protocols in validation phase

In the validation phase, the b value of VN gradient and SMS acceleration rate were predefined based on the results in the optimization phase. We examined 1) the impact of TE on depth-dependent profiles, 2) the BOLD sensitivity in subcortical regions, 3) the robustness of laminar activation profiles, and 4) the feasibility of brain-wide directed functional connectivity (FC).

#### Experiment 2A: Impact of TE on depth-dependent profiles

In conventional 3T fMRI, the typical TE ranges between 30–35 ms (Van Essen et al. 2013). However, to incorporate the VN gradient, the TE in VN fMRI is extended. While the VN gradient mitigates the draining-vein effect, it is noteworthy that the intravascular and extravascular contributions to the BOLD signal also vary with changes in TE (Duong et al. 2003; Kim and Ogawa 2012; Irati Markuerkiaga et al. 2021). To evaluate the impact of this increased TE, a participant underwent multiple finger-tapping task sessions with TEs of 33, 38, and 43 ms, but without the VN gradient. For comparative analysis, the same participant also performed the finger-tapping task with both a TE of 43 ms and the VN gradient. In total, four fMRI sessions were conducted for a single participant: three sessions were conducted without a VN gradient, and one session utilized a VN gradient with a predefined b value of 30 s/mm^2^. All the sessions utilized an SMS factor of 4 and a repetition time (TR) of 3.84 seconds. Each session recorded a total of 282 volumes.

#### Experiment 2B: BOLD sensitivity in subcortical regions

Subcortical regions typically exhibit weaker blood-oxygen-level-dependent (BOLD) responses in comparison to cerebral cortical areas such as the primary motor or somatosensory cortices. This can be attributed to a variety of factors, including a lower SNR and decreased functional specificity. To ensure that the VN gradient does not result in substantial attenuation of subcortical BOLD signals, we investigated the BOLD response within the lateral geniculate nucleus (LGN) using a visual checkerboard task, as outlined in the section of Stimulation Paradigms. Two fMRI sessions were conducted for a single participant, one in the absence of a VN gradient and the other utilizing a VN gradient with a b value of 30. In each session, the participant was presented with twelve stimuli per hemifield, resulting in a total of 24 stimulation blocks. Both sessions utilized an SMS factor of 4 and a repetition time (TR) of 3.84 seconds. A total of 202 volumes were recorded for each session.

#### Experiment 2C: Robustness of laminar activation profile

Six participants were enrolled in this experiment. Each participant underwent two fMRI sessions of finger-tapping task. One in the absence of a VN gradient and the other utilizing a VN gradient with a b value of 30. The sequence of the two sessions was randomized across participants. For each session, a total of 282 volumes were acquired. The SMS factor and TR were the same as those used in Experiment 2A.

#### Experiment 2D: Feasibility of brain-wide directed functional connectivity

Resting-state fMRI data were collected from six participants. Each participant underwent two resting-state fMRI sessions. Participants were instructed to stay motionless, keep their eyes closed, and avoid falling asleep. Only one of the sessions employed the VN gradient with a b value of 30, with the sequence of the two sessions being randomized among participants. The SMS factor and TR were the same as those used in Experiment 2A. For each session, a total of 300 volumes were acquired, taking approximately 19.5 minutes.

### Data Preprocessing

The SMS-accelerated EPI time series were reconstructed using in-house MATLAB code, which performed slice GeneRalized Autocalibrating Partial Parallel Acquisition (slice-GRAPPA) (Setsompop et al. 2012) and in-plane GRAPPA (Griswold et al. 2002) jointly in one step. The reconstructed images were denoised using NORDIC method with estimated g-factor map (function name: ‘NIFTI_NORDIC’). The input images were complex-valued. The width of the smoothing filter for the phase was set as 10, while all other hyperparameters retained their default values. After NORDIC denoising, the images were partial-Fourier reconstructed using a projection-onto-convex-set (POCS) method (Haacke, Lindskogj, and Lin 1991). The time-series data were then motion corrected using FSL, slice-timing corrected using sinc interpolation, and distortion corrected using FSL TOPUP (Smith et al. 2004). For artifact removal, we decomposed the magnitude image of the corrected data into a number of independent components by MELODIC (Beckmann et al. 2005). The noise components were labeled manually and then removed using the FSL command ‘fsl_regfilt’. The time-series signals were band-pass filtered from 0.01 to 0.125 Hz.

### Phase regression

This study implemented the phase regression method for comparative purposes. The phase regression approach was applied to remove the macrovascular component in the BOLD signal as indicated in previous reports (Menon 2002; Knudsen et al. 2023). This macrovascular component not only contributes to alterations in signal magnitude, but also introduces phase variations. By utilizing a linear regression approach between the time series of signal magnitude and phase, the macrovascular-associated contribution to the BOLD signal could be suppressed.

The phase time series underwent the same data processing steps as outlined above except for the step of artifact removal. The phase images of the distortion corrected data were linearly regressed against the time courses of the identified noise components. We employed linear regression for phase images because the FSL command ‘fsl_regfilt’ is incompatible with complex-valued images. Lastly, the filtered BOLD time series of signal magnitude were linearly regressed using the fully-processed and phase-unwrapped time series as the covariate.

### Depth-dependent statistical analysis in volume space

The event-related responses were analyzed using FSL FEAT (M. W. Woolrich et al. 2001; Mark W. Woolrich, Behrens, and Smith 2004). FLAME 1 was used for group-level analysis (Mark W. Woolrich et al. 2004). For the depth-dependent analysis of the finger-tapping task, the M1 area of each individual was identified based on the BOLD activation map. The subsampling along cortical depth can be either volume-based or surface-based. For volume-based subsampling, we generally follow the pipeline suggested by Dr. Huber (https://layerfmri.com/2018/03/11/quick-layering/#more-531). We up-sampled the image slice containing M1 by 5 times, thus achieving an in-plane resolution of 0.18 mm isotropic. The interpolation method utilized was the nearest neighbor. The boundaries between grey matter/white matter (GM/WM) and cerebral spinal fluid/grey matter (CSF/GM) within M1 were manually delineated. With the created M1 label, we employed the LayNii software tool to form 20 equidistant layers (L. R. Huber et al. 2021). The number 20 was determined according to the suggestion by LayNii that the number of layers may be set to at least 4 times larger than the resolution. The depth-dependent functional data were extracted based on the calculated cortical depths. Finally, the depth-dependent profile was computed by averaging the depth-dependent functional data within each layer.

### Surface-based directed functional connectivity analysis

For surface-based subsampling along the cortical depth, we linearly coregistered the individual T1 volume onto the time-averaged EPI volume using Advanced Normalization Tools (ANTs; http://stnava.github.io/ANTs/). Following this, we resampled the processed EPI volumes onto the cortical surface at different cortical depths using the FreeSurfer command ‘mri_vol2surf’. Adapting the layering strategy in volume space, we converted 20 cortical layers from volume to surface space. Here, the cortical depth ranged from −0.125 to 1.0625, with 0 and 1 representing the GM/WM and CSF/GM boundaries, respectively. Surface-based spatial smoothing was applied with full-width half-magnitude (FWHM) of 3 mm.

**Figure 9:**
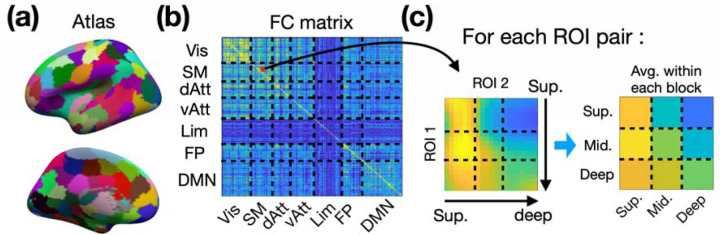
Schematic Overview of Directed Functional Connectivity Calculation Procedure. (a) The surface-based Shen268 functional parcellation. (b) The depth-dependent functional connectivity matrix. (c) The depth-dependent layers were separated into superficial, middle, and deep. The functional connectivity (FC) matrix for each ROI pair was reduced to a simplified 3-by-3 matrix for multiple comparison. Abbreviations: Vis – visual network; SM – sensorimotor network; dAtt – dorsal attention network; vAtt – ventral attention network; Lim – limbic network; FP – frontoparietal network; DMN – default-mode network.

In order to investigate directed functional connectivity throughout the brain, we employed the Shen268 functional parcellation (Shen et al. 2013). The volume-based Shen268 parcellation was converted onto the cortical surface as shown in Figure 9a. The volumetric EPI time-series for each individual were resampled onto their individual brain surfaces before being projected onto the template brain surface. The template surface is of ‘fsaverage5’ from FreeSurfer. We also categorized the parcels in Shen268 atlas into 7 functional networks using the functional atlas reported by Yeo et al. (Thomas Yeo et al. 2011). The time course associated with each region-of-interest (ROI) was obtained by averaging all time courses within the ROI. The depth-dependent functional connectivity was calculated using Pearson correlation and then converted into Fisher’s z values, as illustrated in Figure 9b.

For the purposes of group-level statistical analysis, depth-dependent layers were initially partitioned into superficial, middle, and deep layers, as depicted in the left panel of Figure 9c. Within each block of paired layers, Fisher’s z-values were averaged, thereby reducing the 20-by-20 functional connectivity (FC) matrix for each ROI pair to a simplified 3-by-3 matrix. This size reduction facilitated the management of multiple comparisons. Multiple comparison corrections were performed using the Benjamini & Hochberg False Discovery Rate (FDR) procedure (Benjamini and Hochberg 1995).
